# UGT1A1*28 polymorphism and the risk of toxicity and disease progression in patients with breast cancer receiving sacituzumab govitecan

**DOI:** 10.1002/cam4.70096

**Published:** 2024-08-19

**Authors:** Megan H. Wong, Veronica C. Jones, Wai Yu, Linda D. Bosserman, Sayeh M. Lavasani, Niki Patel, Mina S. Sedrak, Daphne B. Stewart, James R. Waisman, Yuan Yuan, Joanne E. Mortimer

**Affiliations:** ^1^ Department of Medical Oncology & Therapeutics Research City of Hope Comprehensive Cancer Center Duarte California USA; ^2^ Department of Breast Surgery City of Hope Comprehensive Cancer Center Duarte California USA; ^3^ Department of Population Sciences City of Hope Comprehensive Cancer Center Duarte California USA; ^4^ Department of Ambulatory Pharmacy City of Hope Comprehensive Cancer Center Duarte California USA

**Keywords:** metastatic breast cancer, pharmacogenetics, sacituzumab govitecan, SN‐38 toxicity, UGT1A1*28

## Abstract

**Background:**

Sacituzumab govitecan (sacituzumab) emerged as an important agent in metastatic and locally recurrent HER2‐negative breast cancer treatment. UGT1A1 polymorphisms have also been shown to predict sacituzumab toxicity.

**Methods:**

In this retrospective study, we sought to evaluate the associations between UGT1A1 status, toxicity, and therapeutic outcomes in sacituzumab recipients with advanced breast cancer who underwent genotype testing for UGT1A1 alleles (*N* = 68).

**Results:**

We found 17 (25%) of our patients to be homozygous for UGT1A1*28 and 24 (35.3%) were heterozygous. Of seven African American patients with triple‐negative breast cancer, five were homozygous for UGT1A1*28 and two were heterozygous. Patients with a homozygous UGT1A1*28 genotype were significantly more likely to have treatment terminated because of adverse effects. However, the polymorphism was not associated with treatment discontinuation because of disease progression.

**Conclusion:**

This retrospective, real‐world analysis suggests potential clinical utility in UGT1A1 testing for patients receiving sacituzumab, but future trials are needed to confirm the association between genotypes and treatment outcomes.

## 1 INTRODUCTION

Sacituzumab govitecan (sacituzumab) is an antibody‐drug conjugate that has become an important therapeutic option for metastatic and locally recurrent HER2‐negative breast cancers of all subtypes. The drug consists of a monoclonal antibody targeting human trophoblast cell‐surface antigen 2 (Trop‐2) and is linked to the cytotoxic topoisomerase I inhibitor (SN‐38) payload.

Trop‐2 is common in solid tumors, including most triple‐negative breast cancers, and protein overexpression induces rapid cell growth^1^, ^2^, ^3^; continuous cell division results in aggressive disease. Sacituzumab targets Trop‐2, releasing the SN‐38 cytotoxic payload, which is taken up by the bound cell directly and neighboring cells through the bystander effect.^4^ SN‐38 is then degraded by the uridine diphosphate glucuronosyltransferase enzyme coded by the UGT1A1 gene. However, polymorphisms in UGT1A1 are associated with reduced SN‐38 metabolism, resulting in increased toxicities such as diarrhea and neutropenia.^5^, ^6^ We wanted to determine if UGT1A1 polymorphisms are associated with less frequent discontinuations of therapy for progression and increased toxicity in patients with advanced breast cancer undergoing treatment with sacituzumab. The results of the retrospective chart review study are presented here.

## 2 METHODS

Patients with breast cancer treated with sacituzumab were identified through electronic records. The order set for sacituzumab at City of Hope includes an assessment of UGT1A1, which was obtained in the majority of patients. Eligible patients had a primary breast cancer diagnosis, completed UGT1A1 genetic testing, and received at least one dose of sacituzumab. Data abstracted by a single individual (MHW) included demographic information, number of treatment cycles, initial sacituzumab dosing, dose modifications, prior lines of therapy, cancer staging, treatment‐related toxicities, and toxicity management. This study was approved by the City of Hope Institutional Review Board.

Follow‐up began at the first sacituzumab dose. The primary endpoint was sacituzumab discontinuation for disease progression and competing endpoints were discontinuation for toxicity and discontinuation for futility (life expectancy ≤30 days). If the subject moved and became lost to follow‐up without having stopped the drug, observation was censored just before the subject's final dose at our institution.

The study's primary aim was to evaluate whether, among patients with breast cancer treated with sacituzumab, the risk of discontinuation for disease progression was lower with a homozygous UGT1A1*28 genotype than a wild‐type genotype. This hypothesis was tested for statistical significance at *p* < 0.05 in a proportional hazards model for the subdistribution of the primary endpoint, discontinuation for disease progression.^7^ Potential covariates considered in the model were age and body mass index (BMI) at the initiation of sacituzumab, initial dosage (10 mg/kg vs. 7–8 mg/kg), number of prior lines of therapy, triple‐negative disease, and self‐reported ancestry. Covariates were retained in the final model if they improved their fit to the observed data per the Akaike Information Criterion.^8^ The study's secondary aim was to confirm the elevated risk of discontinuation for toxicity in patients with the homozygous UGT1A1*28 genotype, using the same analysis methodology as the primary aim.

## 3 RESULTS

Between July 2020 and September 2022, 67 women and one man met the study inclusion criteria, and their characteristics were analyzed (Table 1). The median age was 57.8 (range 32.3–84.8), and 52 (76.5%) patients had triple‐negative breast cancer. Prior to sacituzumab treatment, all patients had stage IV disease and received a median of 2 (range 1–12) previous breast cancer therapy lines. The most common polymorphism was UGT1A1*28, which was homozygous in 17 (25.0%) patients and heterozygous in 24 (35.3%) patients. There was one person with *1/*37, a less common polymorphism of UGT1A1 that similarly negatively impacts enzymes involved in SN‐38 metabolism.^9^ The remaining 27 (39.7%) patients had the wildtype genotype. During the study period, subjects received a median of 8.5 (range 1–54) doses of sacituzumab and were observed for a median of 3.9 (range 0.9–23.7) months. Fifty‐eight patients started treatment at the standard dose of 10 mg/kg while 10 patients started at reduced doses of 7–8 mg/kg (Table 2).

**TABLE 1:** Patient demographic and clinical characteristics (*N* = 68).

	Median (Range)		*N* (%)
Age in years	57.8 (32.3–84.8)	Ancestry by genotype	
Previous lines of therapy	2 (1–12)	Homozygous	*N* = 17
Doses of SG received	8.5 (1–54)	Non‐Hispanic Caucasian	5 (29.4)
Observation period in months	3.9 (0.9–23.7)	Asian	0 (0)
		Hispanic	7 (41.2)
	*N* (%)	African	5 (29.4)
Sex		Heterozygous^a^	*N* = 24
Female	67 (98.5)	Non‐Hispanic Caucasian	13 (54.2)
Male	1 (1.5)	Asian	3 (12.5)
Triple‐negative breast cancer	52 (76.5)	Hispanic	6 (25)
Initial Dose of SG		African	2 (8.3)
7–8 mg/kg	10 (14.7)	Wild type	*N* = 27
10 mg/kg	58 (85.3)	Non‐Hispanic Caucasian	8 (37.0)
UGT1A1*28 status		Asian	10 (29.6)
Homozygous	17 (25.0)	Hispanic	9 (33.3)
Heterozygous^a^	24 (35.3)	African	0 (0)
Wild type	27 (39.7)		
Self‐reported ancestry			
Caucasian	26 (38.2)		
Asian	13 (19.1)		
Hispanic	22 (32.4)		
African	7 (10.3)		

^a^ Among subjects in the heterozygous category is a single subject whose UGT1A1 mutant allele was *37, corresponding to eight TA repeats rather than the seven repeats in allele *28 or the six TA repeats in the wild type.

**TABLE 2:** Competing risks analysis of the association between UGT1A1*28 homozygosity and discontinuation of sacituzumab govitecan (*N* = 68).

	Discontinuation for disease progression			Discontinuation for toxicity		
	Events/Subjects	Hazards ratio (95% CI)	*p*	Events/Subjects	Hazards ratio (95% CI)	*p*
UGT1A1*28 Genotype						
Homozygous Heterozygous Wild type only	7/17 13/24 18/27	0.80 (0.39–1.65) 0.61 (0.33–1.12) 1.00	0.54 (^a^) 0.12	4/17 0/24 2/27	5.52 (1.15–26.49) 0 1.00	0.03 <0.0001
Initial dosage of sacituzumab govitecan						
7–8 mg/kg 10 mg/kg	3/10 35/38	0.24 (0.07–0.88) 1.00		1/10 5/58	^b^	
Per unit increase in category of body mass index		0.51 (0.34–0.75)			^b^	
From BMI <20 To BMI 20 ≤ 25 To BMI 25+	5/6 17/26 16/36			0/6 2/26 4/36		

^a^ Because it corresponded to the study's single primary hypothesis, this hazards ratio for Disease Progression was the only association appropriate to evaluate for statistical significance.

^b^ The model of Discontinuation for Toxicity was not improved by the inclusion of any covariates, hence no covariate was included in the final model of this secondary endpoint.

Most subjects were followed until discontinuation for disease progression (*n* = 38), toxicity (*n* = 6), or futility (*n* = 4). Patients with futile treatments all subsequently died within 4 weeks of their last dose without documentation of disease progression. Some subjects also became lost to follow‐up (*n* = 3), achieved remission (*n* = 1), or still received sacituzumab at the close of study (*n* = 16). Of those who discontinued treatment due to toxicity, half were hospitalized for febrile neutropenia or pancytopenia, while also having diarrhea or colitis. Beyond the group who discontinued sacituzumab because of intolerance, eight other patients similarly were hospitalized for drug‐induced adverse effects.

All patients self‐reported their race and ethnicity. Relating ancestry and genetic profiles, the wild‐type group was comprised entirely of Asian, non‐Hispanic Caucasian, and Hispanic patients (Table 1). All seven self‐reported African subjects were either carriers or homozygous for UGT1A1*28; none of the African patients included in this study had a wild‐type genotype. Conversely, there were no Asian patients in this single‐center study with a homozygous UGT1A1*28 genotype, and non‐Hispanic Caucasian patients comprised more than half of the heterozygous group.

Univariable analysis did not find an association between homozygous UGT1A1*28 genetic profiles and discontinuation for disease progression. Prior to adjustment for covariates, the hazards ratio was 0.61 (95% confidence interval [CI] 0.26–1.42, *p* = 0.25). Competing risk analysis comparing the homozygous and heterozygous UGT1A1*28 groups with the wild‐type group also showed no significant association between genotype and discontinuation for disease progression. In comparison to the wild‐type group, discontinuation for disease progression in the homozygous UGT1A1*28 group had a hazards ratio of 0.80 (95% CI 0.39–1.65, *p* = 0.54) while that of the heterozygous was 0.61 (95% CI 0.33–1.12, *p* = 0.12). Conversely, there was a significantly increased risk of discontinuation for toxicity in patients homozygous for UGT1A1*28. This was confirmed with a hazards ratio of 5.52 (95% CI 1.15–26.49, *p* = 0.03). We also observed no events of discontinuation for toxicity in patients with a heterozygous genotype, leading to a hazards ratio of 0 (95% CI 0, *p* ≤ 0.0001). However, across our cohort, 76% of patients with a UGT1A1*28 homozygous genotype either started on a lower dose, had a dose reduction, or were intolerant. Treatment alterations and discontinuations for intolerance were observed in 71% and 56% of patients in the heterozygous and wild‐type groups, respectively.

Inclusion of the covariates BMI and initial dosage of sacituzumab improved the model of the primary endpoint. Per unit increase of BMI and the initial dose of sacituzumab at 7–8 mg/kg resulted in hazards ratios of 0.51 (95% CI 0.34–0.75) and 0.24 (95% CI 0.07–0.88), respectively, for discontinuation for disease progression. The other covariates of age, triple‐negative status, line of therapy, and ancestry were excluded from both models for lack of contribution to their fit.

## 4 DISCUSSION

Our exploratory real‐world study in a heavily pretreated population suggests that UGT1A1*28 homozygosity is associated with sacituzumab discontinuation for toxicity. However, we did not identify an association between UGT1A1*28 and discontinuation for progression.

Routine genotyping for UGT1A1 prior to sacituzumab therapy is not recommended in current guidelines to guide drug dosing, despite pharmacogenetic evidence linking UGT1A1 polymorphisms to increased SN‐38 adverse effects.^10^, ^11^ Similar absences in testing recommendations exist with dihydropyrimidine dehydrogenase (DYPD) genotyping for fluoropyrimidine and 5‐fluorouracil chemotherapy, even with research and drug package inserts listing DYPD deficiency as a known risk factor for severe toxicities.^12^, ^13^ As oncologists reconsider the role of routine DYPD screening to reduce toxicities, early UGT1A1 genotyping may similarly identify patients at a high risk for developing toxicities to treatment and inform early dose modifications, increased post‐infusion surveillance, and prophylactic side effect management. Our data suggests value of UGT1A1 testing. However, as our study population was heavily pretreated and did not control for known prognostic factors, future prospective trials are needed to confirm the role of UGT1A1 genotypes on sacituzumab outcomes.

Despite our relatively small sample size, we observed that the prevalence of UGT1A1 polymorphisms varied by patients' ancestry. UGT1A1 is located on chromosome 2q37, which in previous studies has been shown to map strongly to ancestry.^14^, ^15^ Similar to past reports, UGT1A1 polymorphisms were highest in African Americans and lowest in Asian and Polynesian populations.^16^, ^17^, ^18^ Notably, triple‐negative breast cancer is overrepresented in the African American population, and sacituzumab is considered second‐line therapy for this disease.^19^ Considering the relationship between UGT1A1 polymorphisms, increased toxicities, and subsequent therapy discontinuation, it raises the possibility that pharmacogenomics may play a role in the adverse outcomes experienced by African American women with breast cancer. Yet, the ASCENT trial did not contribute to this understanding as 80% of patients enrolled on the sacituzumab arm were Caucasian.^11^ Sacituzumab was initially approved for triple‐negative breast cancer, and all seven African American patients in this study had this subtype, which may explain the overrepresentation of the UGT1A1*28 allele in our cohort. Future studies should investigate the pharmacogenetic relationship between UGT1A polymorphisms, ancestry, and disease outcomes.

Independent of genotype, our study yielded two incidental associations with discontinuation for disease progression. This risk is lower for those initiating sacituzumab below the standard 10 mg/kg dose and for patients with higher BMI. However, the non‐randomized dose assignment and lack of a standardized protocol make the dose‐disease progression association hypothesis‐generating. Additionally, the observed benefit for greater BMI suggests sacituzumab's dosing may be more optimal for overweight and obese patients. However, further research is needed to explore the relationships between dosing, BMI, and sacituzumab efficacy.

These findings have the usual limitations of a retrospective study. Our sample size provides an adequate (80%) power to detect a marked association, specifically a hazards ratio below 0.33 or above 3.0. We support the relative risk of the homozygous UGT1A1*28 genotype for treatment discontinuation for toxicity which is consistent with the outcomes observed in the ASCENT safety clinical trial.^11^ Additionally, our single‐center ancestry‐genotypic frequencies for UGT1A1 in sacituzumab recipients with breast cancer align with past studies that investigated the prevalence of UGT1A1*28 across races and ethnicities.^16^, ^17^, ^18^

Genomics have become increasingly important in the treatment planning of all stages of breast cancer, and pharmacogenetics should be considered equally important. As guidance for the adoption of DYPD screening for fluoropyrimidine and 5‐fluorouracil therapies is being reassessed, we support a similar reevaluation of the pharmacogenetics guidelines for sacituzumab administration.

## AUTHOR CONTRIBUTIONS

**Megan H. Wong:** Conceptualization (equal); data curation (lead); investigation (equal); methodology (equal); writing – original draft (lead); writing – review and editing (equal). **Veronica C. Jones:** Writing – review and editing (equal). **Wai Yu:** Conceptualization (equal). **Linda D. Bosserman:** Investigation (equal). **Sayeh M. Lavasani:** Investigation (equal). **Niki Patel:** Investigation (equal). **Mina S. Sedrak:** Investigation (equal). **Daphne B. Stewart:** Investigation (equal). **James R. Waisman:** Investigation (equal). **Yuan Yuan:** Investigation (equal). **Joanne E. Mortimer:** Conceptualization (equal); investigation (equal); methodology (equal); writing – original draft (supporting); writing – review and editing (equal).

## FUNDING INFORMATION

This study was supported by the Cancer Center Support Grant P30CA033572 from the National Cancer Institute of the National Institutes of Health.

## CONFLICT OF INTEREST STATEMENT

YY serves as a consultant for Imugene and Gilead Sciences, is on the speakers bureau for Merck, Gilead Sciences, AstraZeneca, and Daiichi Sankyo, and received a grant from Merck. JEM serves as a consultant with GE HealthCare and provides research support for AstraZeneca. All other authors have declared no competing interests for this work.

## ETHICS STATAMENT

This study was reviewed and approved by the City of Hope Institutional Review Board.

## PATIENT CONSENT

The City of Hope Institutional Review Board approved the waiver of written informed consent for this retrospective study.

## Data Availability

Research data are not shared.
